# Manganese Exposure: Delayed Effects and Biomarkers in adult male and female Wistar rats

**DOI:** 10.1007/s12035-026-05812-0

**Published:** 2026-03-30

**Authors:** Tuany Eichwald, Diego Perinetto, Valéria Zardo, Karina Giacomini Varela, Analu Mantovani, Antuani Rafael Baptistella, Diego de Carvalho, Aline Pertile Remor

**Affiliations:** 1 Life Sciences Area, Postgraduate Program in Bioscience and Health (PPGBS), University of Western Santa Catarina (UNOESC), Joaçaba, SC 89600-000 Brazil; 2 Department of Biomedical and Molecular Sciences, Queen’s University, Kingston, Canada; 3 Biological Sciences Degree, Life Sciences Area, University of Western Santa Catarina (UNOESC), Joaçaba, SC Brazil; 4 Soil Laboratory, University of Western Santa Catarina (UNOESC), Campos Novos, SC Brazil

**Keywords:** Manganese neurotoxicity, Delayed effects, Oxidative and mitochondrial dysfunction, Non-invasive biomarkers, Sex-dependent differences

## Abstract

Manganese (Mn) is an essential trace element required for various physiological processes, but excessive exposure can lead to neurotoxicity and motor impairments. This study investigated the immediate and delayed behavioral effects and potential tissue biomarkers, as well as the immediate oxidative and metabolic alterations, following subacute Mn intoxication in adult male and female Wistar rats. Animals received MnCl₂ (15 mg/kg, intraperitoneally, for 5 days per week over 4 weeks, totaling 30 days of exposure). Following this treatment, animals were allocated into two independent experimental endpoints. The first group was evaluated immediately after the end of the 30-day MnCl_2_ exposure period. The second group underwent an additional 30-day Mn-free recovery period before evaluation, resulting in a total experimental duration of 60 days for this group. Corresponding control groups were run in parallel for both endpoints. After the exposure period, no significant motor coordination deficits were observed. However, motor impairments emerged after the recovery period, suggesting delayed neurotoxic effects. Oxidative stress markers revealed decreased non-protein thiol levels in the cortex and hippocampus, indicating Mn-induced antioxidant depletion, while mitochondrial complex activities varied by sex and brain regions. Although Mn concentrations in brain tissues returned to baseline after recovery, elevated Mn levels persisted in nails and teeth, suggesting that these tissues are promising non-invasive biomarkers of cumulative Mn exposure. These findings demonstrate that, within a predefined two-endpoint design, Mn neurotoxicity manifests in a time- and sex-dependent manner, highlighting distinct temporal patterns of functional and biochemical vulnerability. Thus, the identification of nails and teeth as reliable non-invasive biomarkers further underscores the complexity of Mn toxicity and highlights the importance of considering sex-specific responses in toxicological assessments.

**Graphical Abstract:**

Summary of sex-dependent behavioral, biochemical, and bioaccumulation outcomes following subacute manganese (Mn) exposure in adult Wistar rats. Male and female rats were exposed to MnCl₂ (15 mg/kg/day, intraperitoneally) for 30 consecutive days and evaluated at two predefined experimental endpoints: immediately after the exposure period and after an additional 30-day Mn-free recovery interval. Immediate assessments revealed sex-dependent alterations in anxiety-like behavior and region-specific oxidative and mitochondrial changes. After the recovery period, delayed motor coordination impairments were evident, particularly in males, despite normalization of brain Mn levels. Mn bioaccumulation persisted in peripheral tissues, including hair, nails, and teeth, in both sexes. Overall, the results show that, within this two-endpoint design, Mn induces time-dependent and sex-specific neurotoxic effects, with nails and teeth emerging as reliable non-invasive biomarkers of cumulative Mn exposure.

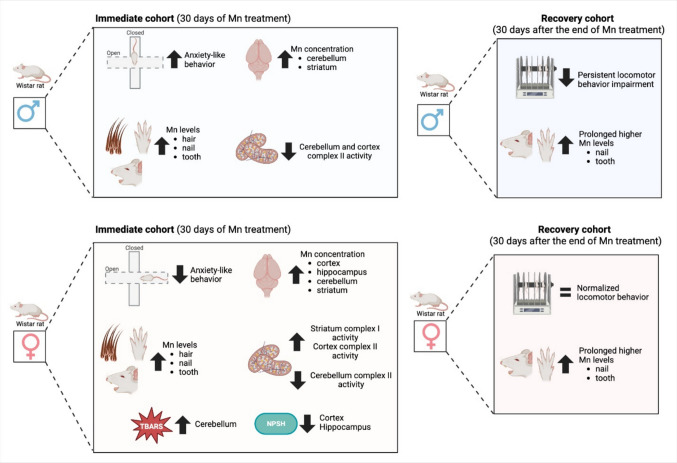

**Supplementary Information:**

The online version contains supplementary material available at 10.1007/s12035-026-05812-0.

## Introduction

Manganese (Mn) is an essential trace element required for multiple physiological processes, but when present in excess it becomes neurotoxic [1]. Toxic Mn accumulation may arise from genetic disorders, secondary to liver dysfunction, and/or environmental exposure exceeding the World Health Organization's recommended intake (0.7–10.9 mg/day), including contaminated drinking water or pesticide use [2–4].

The brain is particularly vulnerable to Mn excess, with preferential accumulation in basal ganglia structures, such as the striatum, substantia nigra, and globus pallidus. The clinical manifestations, collectively referred to as manganism, resemble Parkinsonian syndromes and include motor, neuropsychological, and cognitive deficits [4–9]. Importantly, adverse effects have also been reported in both humans [10, 11] and animal experimental models [12, 13], even at low-level exposure [6]. These effects can be modulated by factors such as age, sex, and ethnicity [14].

Although the mechanisms underlying Mn neurotoxicity are not fully defined, several lines of evidence implicate oxidative stress, mitochondrial dysfunction, neuroinflammation, and neurotransmitter disruption as central pathways contributing to neural injury [15–22].

Despite substantial evidence of Mn-induced neurotoxicity, few studies have systematically evaluated whether metabolic, biochemical, and long-term neurobehavioral alterations persist after exposure cessation or recover over time. Moreover, the potential utility of peripheral keratinized tissues, such as nails and teeth, as cumulative and non-invasive biomarkers of Mn exposure remains poorly understood. To address these gaps, we employed an in vivo model in adult male and female Wistar rats exposed to MnCl₂ for 30 days, using two predefined experimental endpoints: one cohort evaluated immediately at the end of exposure and a second cohort evaluated after an additional 30-day Mn-free recovery interval. This design enabled us to distinguish immediate from delayed effects on behavior, brain region-specific oxidative and mitochondrial alterations, and peripheral Mn bioaccumulation, allowing the characterization of sex-dependent vulnerabilities and the identification of reliable biomarkers of cumulative Mn exposure.

## Material and Methods

### Animals

Male and female adult Wistar rats were obtained from the Federal University of Rio Grande do Sul (UFRGS), Brazil, and acclimated to the housing conditions for 20 days upon arrival. Adult rats (8 weeks of age; body mass 200–300 g) were housed in the UNOESC animal facility under controlled conditions (22 ± 1 °C; 12-h light/dark cycle; 30–70% humidity). Animals had ad libitum access to water and standard chow containing 110 mg/kg of Mn. The experimental protocol was approved by the UNOESC Ethics Committee for Animal Research (protocol number 45/2016). All efforts were made to minimize the number of animals used and to reduce animal suffering.

For this study, a total of 80 adult Wistar rats were used. Animals were prospectively allocated to two predefined experimental cohorts (immediate and recovery), each initially comprising *n* = 10 animals per sex per treatment group. Detailed allocation of animals across behavioral and biochemical endpoints is provided in Supplementary Table S1.

### Chemicals

All reagents were of analytical grade or the highest purity available for pharmaceutical applications. Manganese chloride tetrahydrate (MnCl₂·4H₂O, MW 197.91) was obtained from Fisher Chemical (Fair Lawn, NJ). Nitric acid was purchased from VWR International (Radnor, PA), and perchloric acid was obtained from RICCA Chemical Company (Arlington, TX). Anhydrous disodium hydrogen phosphate (Na₂HPO₄), sodium phosphate monobasic (NaH₂PO₄), bovine serum albumin (BSA), ethylenediaminetetraacetic acid (EDTA), and sucrose were purchased from Sigma-Aldrich (St. Louis, MO). Manganese solutions were freshly prepared by dissolving MnCl₂·4H₂O in sterile 0.9% saline to achieve concentrations of 15 mg/kg. Solutions were prepared every two weeks and stored at 4 °C. Prior to intraperitoneal (i.p.) injection, the solutions were equilibrated to room temperature for 30 min.

### Mn Treatment In Vivo

**Scheme 1:**
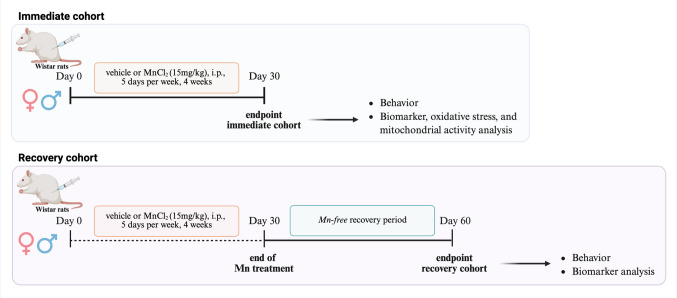
Experimental design and timeline of subacute manganese exposure and evaluation endpoints. Adult male and female Wistar rats received MnCl₂ (15 mg/kg, i.p.) once daily, five days per week, for four consecutive weeks. From the beginning of the study, animals were allocated into two predefined experimental endpoints. The immediate cohort was evaluated and euthanized at the end of the 30-day exposure period to assess early behavioral outcomes, brain region–specific oxidative and mitochondrial alterations, and Mn/Fe tissue accumulation. In parallel, the recovery cohort was maintained for an additional 30-day Mn-free recovery interval before evaluation (totaling 60 days), allowing the assessment of delayed behavioral effects, along with Mn and Fe levels in brain and peripheral tissues. Control groups were run in parallel for each endpoint and received vehicle injections. Behavioral tests, biochemical analyses, and metal quantification were performed according to the timeline indicated

The subacute Mn experimental intoxication protocol was adapted from O’Neal et al. [23]. Animals were randomly assigned to two predefined experimental endpoints groups to distinguish immediate from delayed effects of Mn exposure. All treated animals received MnCl_2_ (15 mg/kg, intraperitoneally (i.p)), once daily for five consecutive days per week over four weeks (30 days) to induce intoxication. Both cohorts underwent the same dosing regimen; however, the recovery cohort was maintained for an additional 30-day Mn-free period to assess delayed neurotoxic outcome (totaling 60 days). A matched control group for each endpoint was run in parallel and received i.p. injections of the vehicle (1 mL/kg of 0.9% sterile saline) (Scheme 1).

### Behavioral tests

#### Elevated Plus-maze

Anxiety-related behaviors were assessed separately in animals evaluated immediately after exposure and in those evaluated after a 30-day Mn-free recovery period using the elevated plus-maze (EPM). The EPM is a plus-shaped wooden apparatus consisting of two opposite open arms (50 cm × 10 cm) and two opposite enclosed arms (50 cm × 10 cm × 40 cm) extending from a central platform (10 cm × 10 cm). The maze is elevated 50 cm above the floor. Each animal was individually placed in the center of the maze, facing one of the enclosed arms, and allowed to explore freely for 5 min [24]. The following parameters were recorded: the frequency of open-arm entries, the frequency of enclosed-arm entries, and the time spent in open arms. An entry was defined when the animal placed all four paws into an arm. A decrease in open-arm entries and/or time spent in the open arms was interpreted as an anxiogenic effect. Final sample sizes for behavioral analyses are provided in Supplementary Table S2.

#### Rotarod

Motor coordination and balance were assessed separately in animals evaluated immediately after exposure and in those evaluated after a 30-day Mn-free recovery period using an accelerating rotarod apparatus (Insight Scientific Equipments, Ribeirão Preto, SP, Brazil). The rotarod consists of a grooved metal roller, 6 cm in diameter and 16 cm in height, divided into 9 cm wide compartments. During the adaptation phase, animals were placed on the non-rotating roller until they could maintain a stable position with all four paws for 30 s. To ensure the animals' aptitude for the task, a baseline test followed, where the rats were placed on the rotarod at a constant speed of 12 rpm for one min. Motor coordination was evaluated using an accelerated protocol in a single session, where the rotation speed incrementally increased from 0 to 40 rpm over a span of 300 s. The latency to fall, recorded in seconds, was measured [25]. Final sample sizes for behavioral analyses are provided in Supplementary Table S2.

### Tissue Preparation for Analysis

At each experimental endpoint (immediate evaluation after 4 weeks of Mn exposure or recovery evaluation following 4 weeks Mn-free recovery period), the animals were anesthetized with ketamine (80 mg/kg) and xylazine (12 mg/kg) and euthanized by decapitation. Brain tissues and serum were rapidly collected and stored at − 80 °C for further analyses. Additionally, samples of hair, nails, and teeth were immediately collected and prepared for subsequent analysis.

### Determination of Mn and/or Fe by Atomic Absorption Spectrometry

Mn and Fe concentrations were analyzed in animals from both endpoints. 100 mg of brain tissue, 50 mg each of hair and teeth, 20 mg of nails, and 300 μL of serum [26], were used. Tissues were digested in a 4:1 mixture of 65% nitric acid and perchloric acid for 24 h at room temperature. After digestion, samples were placed in an ultrasonic bath at 70 °C for 3 h, followed by the addition of 3.5 mL of 1% perchloric acid. The final solution was filtered through a 0.22 μm syringe filter. Metal concentrations were determined using a Perkin Elmer Atomic Absorption Spectrometer (Analyst 800®) calibrated with standard curves. Results were expressed as μg/g for solid tissues and μg/mL for serum.

### Oxidative and Metabolic Parameters

Oxidative and metabolic parameters were assessed in brain tissues collected from animals in the immediate endpoint cohort and were homogenized in 10 volumes of 20 mM HEPES buffer (pH 7.4) and centrifuged at 5,000 × *g* for 10 min at 4 °C. The supernatant was used to determine thiobarbituric acid-reactive substances (TBARS) and non-protein thiol (NPSH) levels.

#### Measurement of Thiobarbituric Acid-reactive Substances (TBARS)

Lipid peroxidation was determined in the immediate endpoint cohort, using the TBARS method, as described by Esterbauer and Cheeseman [27]. Briefly, brain tissue homogenates were mixed with an equal volume of 10% trichloroacetic acid (TCA) and centrifuged at 3,000 × g for 10 min. An aliquot of the supernatant was incubated with an equal volume of 0.67% thiobarbituric acid (TBA) in 7.1% sodium sulfate and heated in a water bath for 45 min. The absorbance of the pink complex formed by the reaction of TBA with lipid peroxidation by-products was measured at 535 nm using a Synergy HTX microplate reader (BioTek®). A standard calibration curve was prepared with malondialdehyde (MDA) standards treated identically to the samples. TBARS levels were expressed as nmol MDA/mg of protein.

#### Non-protein Thiol Groups (NPSH) Measurement

NPSH groups, primarily glutathione (GSH), were determined in the immediate endpoint cohort, using Ellman’s reagent [28]. Previously prepared brain tissue supernatants were treated with an equal volume of 10% TCA and centrifuged. Aliquots of the supernatant were diluted in 80 mM sodium phosphate buffer (pH 7.4). To these diluted samples, 0.5 mM DTNB (5,5'-dithiobis-2-nitrobenzoic acid) was added. Absorbance of the colorimetric reaction between DTNB and thiol groups was measured at 412 nm using a Synergy HTX microplate reader (BioTek®) after a 10-min reaction. NPSH levels were expressed as nmol NPSH/mg of protein.

### Mitochondrial Complex I and II Activities

Brain tissues from the immediate endpoint cohort were homogenized in 10 volumes of 4.4 mM potassium phosphate buffer (pH 7.4) containing 0.3 M sucrose, 5 mM MOPS, 1 mM EGTA, and 0.1% bovine serum albumin. Homogenates were centrifuged at 3,000 × *g* for 10 min at 4 °C. The supernatant was used to measure mitochondrial complex I and II activities, following the methods of Latini et al. [29] [29]. Complex I activity was determined by measuring NADH-dependent ferricyanide reduction at 420 nm [30]. Complex II activity (succinate–2,6-dichloroindophenol (DCIP) oxidoreductase) was measured as described by Fischer et al. [31]. Enzyme activities of the respiratory chain complexes were expressed as nmol/min/mg of protein, measured at 37 °C using a Synergy HTX microplate reader (BioTek®).

### Protein Determination

Protein concentration was measured by Lowry method [32] using bovine serum albumin as the standard.

### Statistical Analysis

Data are presented as mean ± standard error of mean (SEM). Body weight gain was analyzed using two-way repeated-measures ANOVA followed by Tukey’s post hoc test. Data from behavioral parameters and biochemical determinations were analyzed using one-way or two-way ANOVA, to verify the interaction between group and sex, both followed by Tukey’s post hoc test when *p* was significant. The “n” values represent the number of animals included in each test. Only significant *p* values were given in the text. Differences between groups and sexes were considered significant when *p* < 0.05. Statistical analyses were performed using the Statistica® version 9.0 software and the graphics were made using GraphPad Prism® software version 10 (GraphPad Software, San Diego, CA, USA).

Regarding sample size considerations, all groups initially comprised *n *= 10 animals per sex per treatment; however, subsets of animals were allocated to specific endpoints according to predefined methodological requirements, resulting in variation in final n across behavioral and biochemical analyses. Importantly, group sizes were balanced within each individual analysis. Two-way ANOVA models (including repeated-measures when applicable) were applied, and all relevant interaction terms were explicitly tested. Pearson’s correlation analyses were performed to evaluate associations between peripheral Mn levels and oxidative parameters. Detailed sample sizes per outcome are provided in Supplementary Table S1, significant interaction analyses are summarized in Supplementary Table S2, and correlation results are presented in Supplementary Table S3.

## Results

**Fig. 1:**
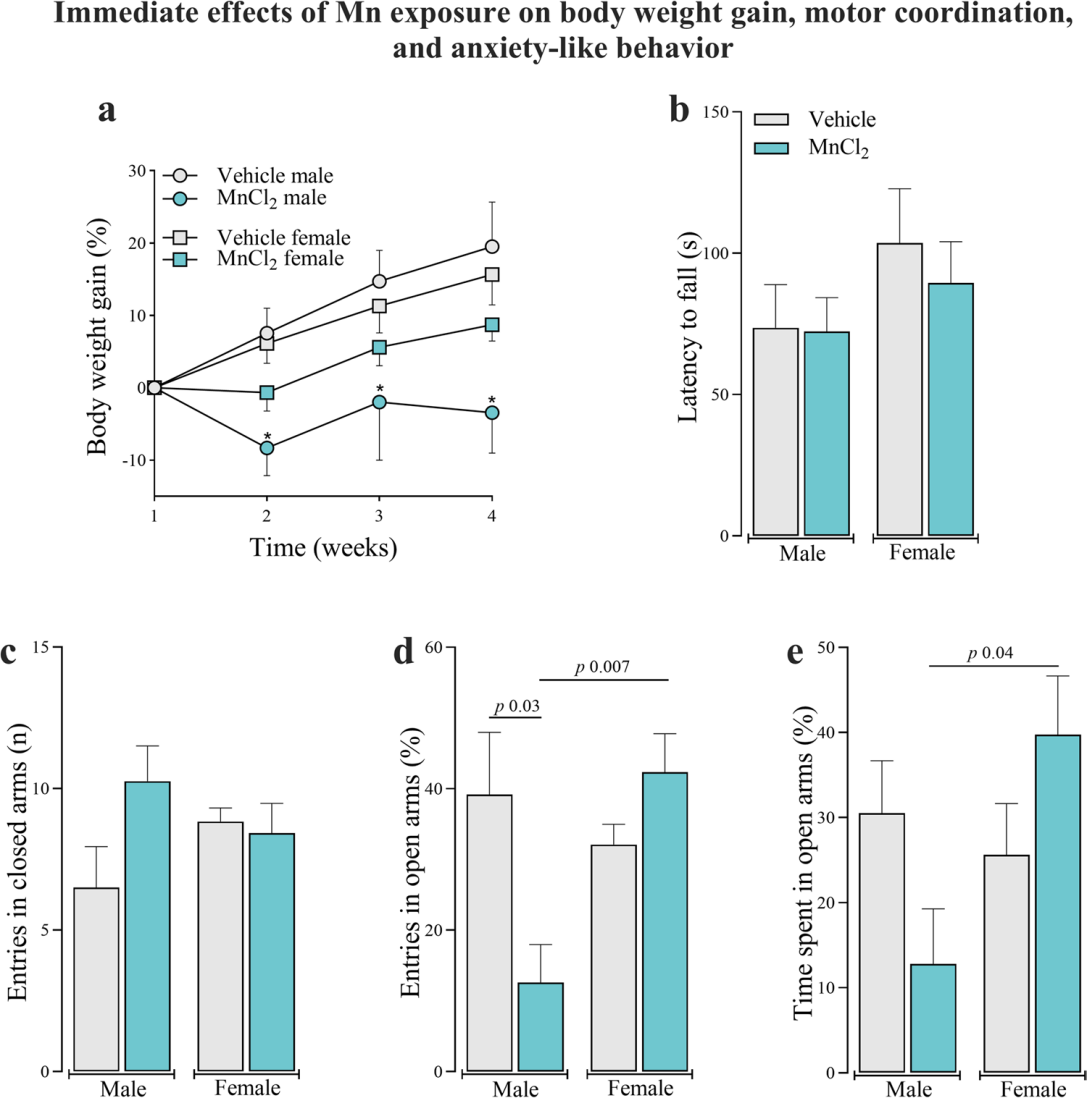
Immediate effects of subacute Mn exposure (MnCl_2_; 15 mg/kg i.p.; daily for 5 days/4 weeks) on body weight gain (**a**), motor coordenation (**b**) and anxiety-like behavior (**c**-**e**) in adult male and female Wistar rats. Data are expressed as mean ± standard error of the mean (10 animals/group for body weight gain and 6 animals/group for behavioral tests). Significant *p*-values are shown in each panel. For panel (**a**) ^∗^*p* < 0.05 when compared to vehicle group between weeks of treatment using Two-way ANOVA for repeated measures followed by Tukey post-hoc test. For panel (**b**-**e**) one and Two-way ANOVA followed by Tukey post-hoc test were used for behavioral comparisons

Figure 1 illustrates the immediate effects of subacute Mn exposure, evaluated at the end of the 30-day treatment period, on body weight gain, motor coordination, and anxiety-like behavior. Compared to male controls, Mn exposure significantly reduced body weight gain in male rats during weeks 2, 3, and 4 of the treatment period. In contrast, no significant differences were observed in female, regardless of Mn treatment (Fig. 1a) (interaction between group and week: [F_(1,19)_ = 32.625; *P* < 0.001], interaction between group and sex: [F_(1,19)_ = 62.997; *P* < 0.001] and interaction between group, sex and week: [F_(1,19)_ = 8.66; *P* < 0.001]). Given that Mn intoxication causes Parkinson-like symptoms, motor coordination was evaluated using the Rotarod test. No differences in latency to fall were observed between Mn-treated and control animals of either sex in the immediate post-exposure assessment (Fig. 1b). Based on these observations, we next assessed whether Mn exposure alters anxiety-like and exploratory activity using the EPM test. No differences were observed in the number of entries into the closed arms of the EPM in the immediate post-exposure cohort (Fig. 1c). Mn-exposed males showed a significant lower percentage of open-arm entries compared to control males (Fig. 1d) [F_(3,19)_ = 5.049; *P* < 0.01], indicating increased anxiety-like behavior. This effect was not observed in females. Additionaly, Mn-treated females exhibited significantly higher percentage of open-arm entries compared to Mn-treated males (Fig. 1d**)** (interaction between group and sex: [F_(1,19)_ = 10.03; *P* < 0.01]). Similarly, Mn-treated females spent more time in the open arms compared to Mn-treated males after the 30-day treatment period (Fig. 1e) (interaction between group and sex: [F_(1,19)_ = 6,13; *P* < 0.05]).

**Fig. 2:**
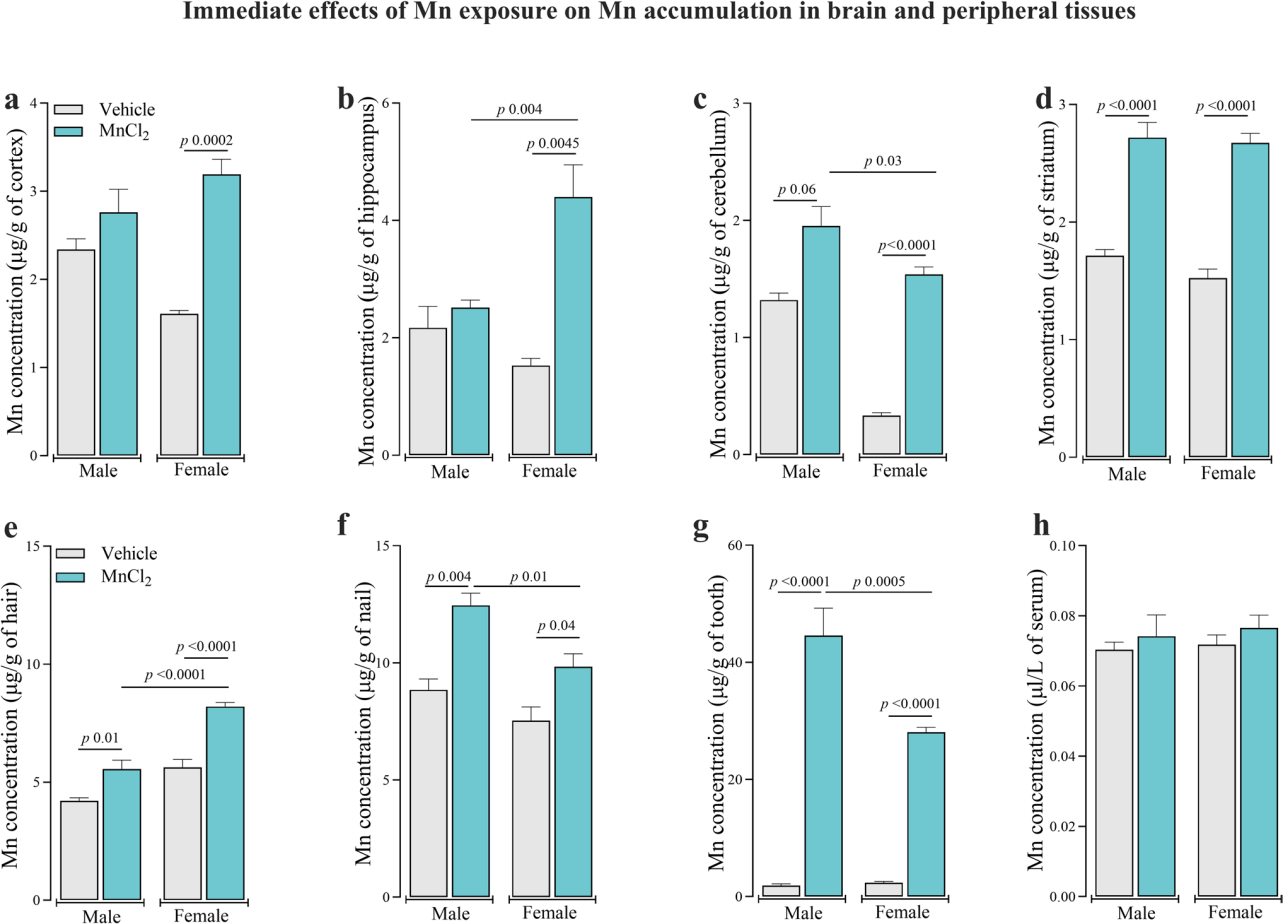
Immediate effect of subacute Mn exposure (MnCl_2_; 15 mg/kg i.p.; daily for 5 days/4 weeks) on Mn concentrations in the cerebral cortex (**a**), hippocampus (**b**), cerebellum (**c**), striatum (**d**), hair (**e**), nail (**f**), tooth (**g**) and serum (**h**) in adult male and female Wistar rats. Data represent mean ± standard error of the mean (6 animals/group). Significant *p*-values and interaction effects are indicated in each panel. One and two-way ANOVA followed by Tukey post-hoc test was used for statistical comparisons

Mn accumulation in the central nervous system (CNS) is associated with motor impairment. To determine whether Mn preferentially accumulated in specific brain structures during the immediate post-exposure period, Mn concentrations were quantified in the cortex, hippocampus, cerebellum and striatum (Fig. 2a-d). In females, Mn exposure significantly increased Mn concentration in the cortex (Fig. 2a) [F_(3,24)_ = 9.406; *P* < 0.001], hippocampus [F_(3,24)_ = 10.38; *P* < 0.001] (Fig. 2b), cerebellum [F_(3,25)_ = 29.81; *P* < 0.001] (Fig. 2c), and striatum [F_(3,25)_ = 33.1; *P* < 0.001]) (Fig. 2d). In males, Mn accumulation was detected primarily in the striatum (Fig. 2d) and, although not statistically significant, a trend toward increased Mn levels was observed in the cerebellum (p = 0.06) (Fig. 2c). Thus, two-way ANOVA confirmed sex-dependent Mn deposition patterns in the hippocampus (Fig. 2b) and cerebellum (Fig. 2c) of Mn treated groups (interaction between group and sex: hippocampus [F_(1,24)_ = 9.478; *P* < 0.01] and cerebellum [F_(1,24)_ = 4.911; *P* < 0.05]).

Given the well-established correlation between iron (Fe) and Mn homeostasis, Fe concentrations were assessed in the same brain regions at both predefined experimental endpoints, immediate after the exposure period and after the 30-day Mn-free recovery period (Suppl. Figure S1). Our analysis revealed that during the immediate post-exposure assessment cohort, Mn exposure let to a significantly lower Fe concentration in the striatum of females when comparing to their controls [F_(3,24)_ = 4.375; *P* < 0.05]) (Suppl. Fig. S1d). Sex-dependent patterns were also evident, with females showing reduced Fe levels in the hippocampus (Suppl. Fig. S1b) and increased Fe levels in the cerebellum (Suppl. Fig. S1c) relative to Mn-treated males (interaction between group and sex: hippocampus [F_(1,25)_ = 4.306; *P* < 0.05] and cerebellum [F_(1,25)_ = 12.53; *P* < 0.05]). In contrast, after the 30-day Mn-free recovery period, Fe concentration did not differ between Mn-treated and controls animals in any brain region (Suppl. Fig. S1e-h), suggesting normalization of Fe homeostasis over time despite the early sex-specific disruptions observed immediately after exposure.

To evaluate the usefulness of peripheral tissues as biomarkers of systemic Mn exposure, Mn concentrations were measured in hair, nails, teeth, and serum, during the immediate post-exposure assessment (Fig. 2e-h). Mn exposure led to a significant increase in Mn deposition in the hair (Fig. 2e) [F_(3,21)_ = 55.16; *P* < 0.001], nails (Fig. 2f) [F_(3,21)_ = 10.29; *P* < 0.001], and teeth (Fig. 2g) [F_(3,20)_ = 76.51; *P* < 0.01] in both male and females in comparison to their controls. Serum Mn did not differ between groups (Fig. 2h). Furthermore, Mn-treated females exhibited significantly higher Mn concentrations in hair compared to males (Fig. 2e). In contrast, males showed higher Mn levels in nails (Fig. 2f) and teeth (Fig. 2g) relative to females, indicating tissue-specific and sex-dependent patterns of Mn deposition (interaction between group and sex: hair [F_(1,21)_ = 5.594; *P* < 0.001], nails [F_(1,21)_ = 1.149; *P* < 0.05] and tooth [F_(1,20)_ = 12.748; *P* < 0.001]).

**Fig. 3:**
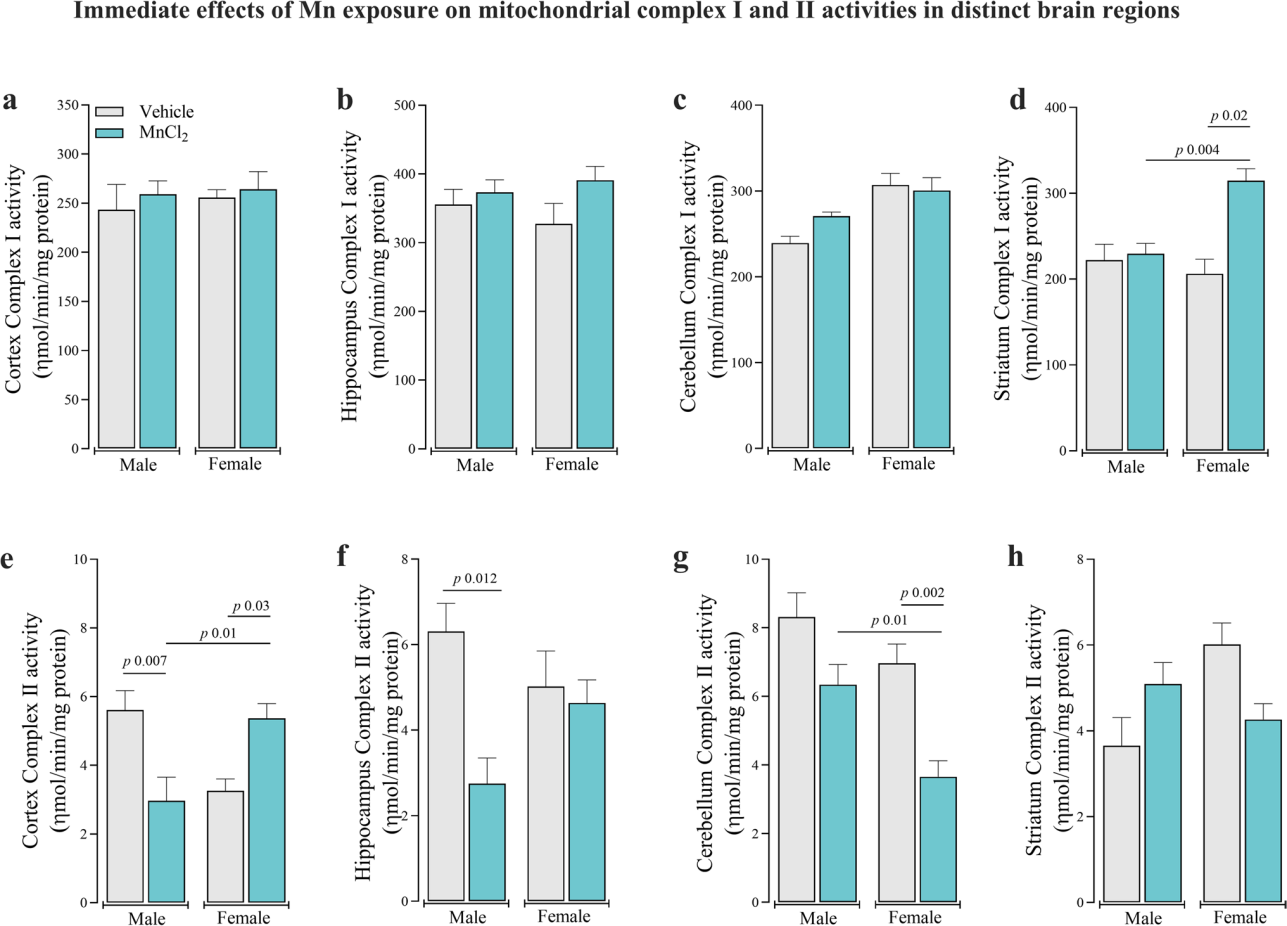
Immediate effect of subacute Mn exposure (MnCl_2_; 15 mg/kg i.p.; daily for 5 days/4 weeks) on mitochondrial respiratory chain complex I activity in the cerebral cortex (**a**), hippocampus (**b**), cerebellum (**c**), and striatum (**d**), and on complex II activity in the cerebral cortex (**e**), hippocampus (**f**), cerebellum (**g**), and striatum (**h**) in adult male and female Wistar rats. Data represent mean ± standard error of the mean (7 animals/group). Significant *p*-values are described in each panel. One and Two-way ANOVA followed by Tukey post-hoc test was used for statistical comparisons

To further characterize Mn-induced metabolic alterations, immediate post-exposure activities of mitochondrial respiratory chain complexes I (CI) and II (CII) were measured across brain regions (Fig. 3). CI activity was selectively altered in a sex- and region- dependent manner, with a significant increased in the striatum of Mn-treated females compared to their Mn-treated counterparts [F_(3,27)_ = 9.849; *P* < 0.05] and to Mn-treated males (Fig. 3d) (interaction between group and sex: [F_(1,27)_ = 10.248; *P* < 0.001]). No differences were detected in the other brain regions. Regarding CII activity, distinct and sex-dependent immediate effects of Mn exposure were observed across brain regions (Fig. 3e-h). Mn-treated males exhibited a significant inhibition of cortical CII activity, whereas Mn-treated females showed a significant increased compared to their respective controls [F_(3,23)_ = 7.323; *P* < 0.01], resulting in a robust different sex-pattern (interaction between group and sex: [F_(1,23)_ = 21.829; *P* < 0.01]) (Fig. 3e). Furthermore, Mn exposure let to a significant reduction in CII activity in the male hippocampus [F_(3,23)_ = 4.534; *P* < 0.05] and female cerebellum [F_(3,24)_ = 11.09; *P* < 0.01] when compared to the controls (Fig. 3f and g). No significant alterations in CII activity were detected in the striatum (Fig. 3h).

**Fig. 4:**
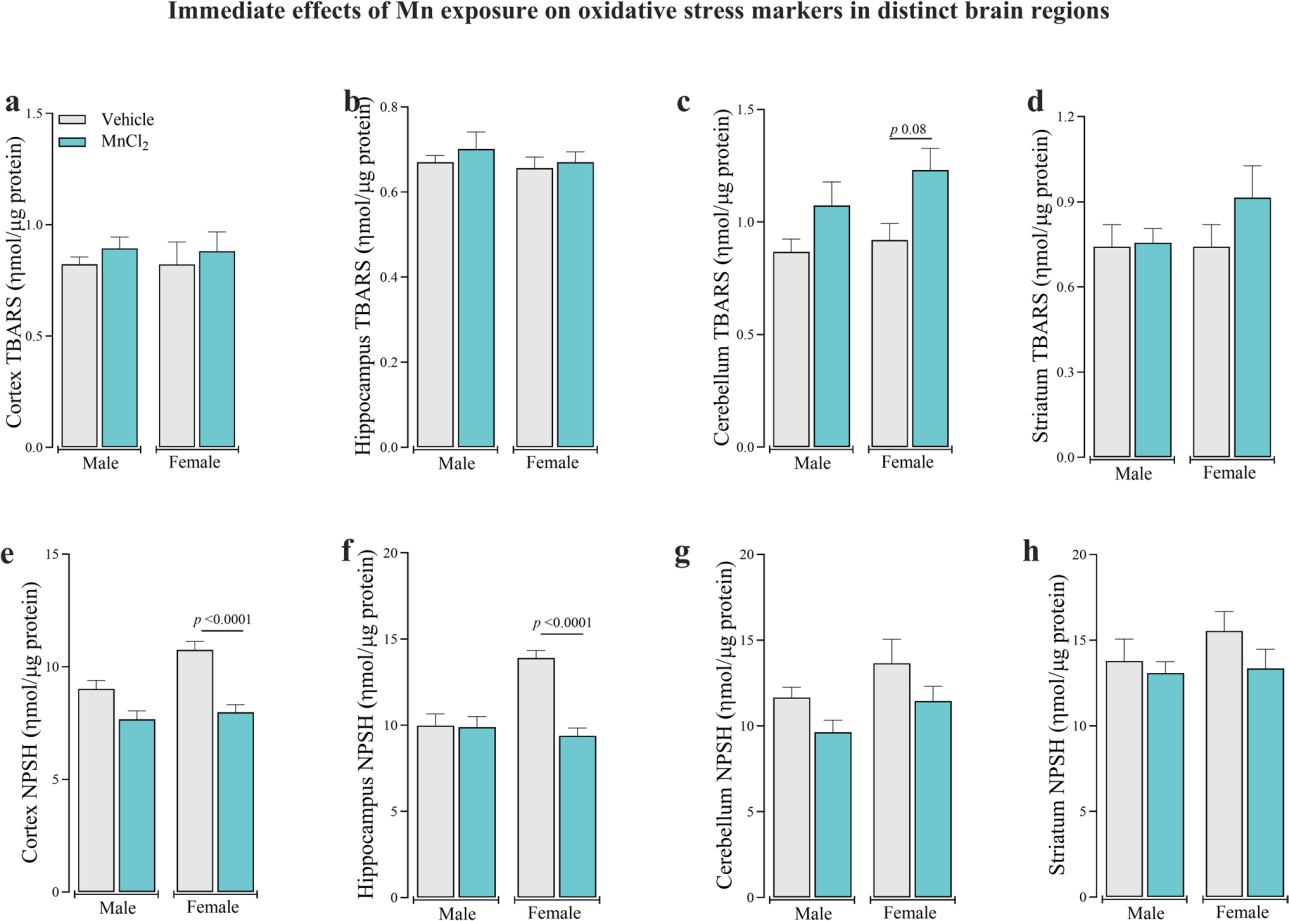
Immediate effects of subacute Mn exposure (MnCl_2_; 15 mg/kg i.p.; daily for 5 days/4 weeks) on lipid peroxidation (TBARS) levels in the cerebral cortex (**a**), hippocampus (**b**), cerebellum (**c**), and striatum (**d**), and on non-protein thiol (NPSH) levels in the cerebral cortex (**e**), hippocampus (**f**), cerebellum (**g**), and striatum (**h**) in adult male and female Wistar rats. Data represent mean ± standard error of the mean (9 animals/group). Significant *p*-values are described in each panel. One and Two-way ANOVA followed by Tukey post-hoc test was used for statistical comparisons

Given the observed alterations in mitochondrial activity in the immediate post-exposure cohort, oxidative stress markers were assessed at the end of the 30-day Mn exposure period (Fig. 4). Lipid peroxidation analysis demonstrated no significant differences in TBARS levels between Mn-treated and control animals across any brain region (Fig. 4a-d). In contrast, the NPSH levels were significantly decreased in the cortex [F_(3,36)_ = 13.17; *P* < 0.001] and hippocampus [F_(3,36)_ = 13.33; *P* < 0.001] of Mn-treated females compared to controls (Fig. 4e and f). No NPSH content alterations were observed in the cerebellum (Fig. 4g) or striatum (Fig. 4h). These findings suggest selective oxidative vulnerability in specific brain regions following Mn exposure.

**Fig. 5:**
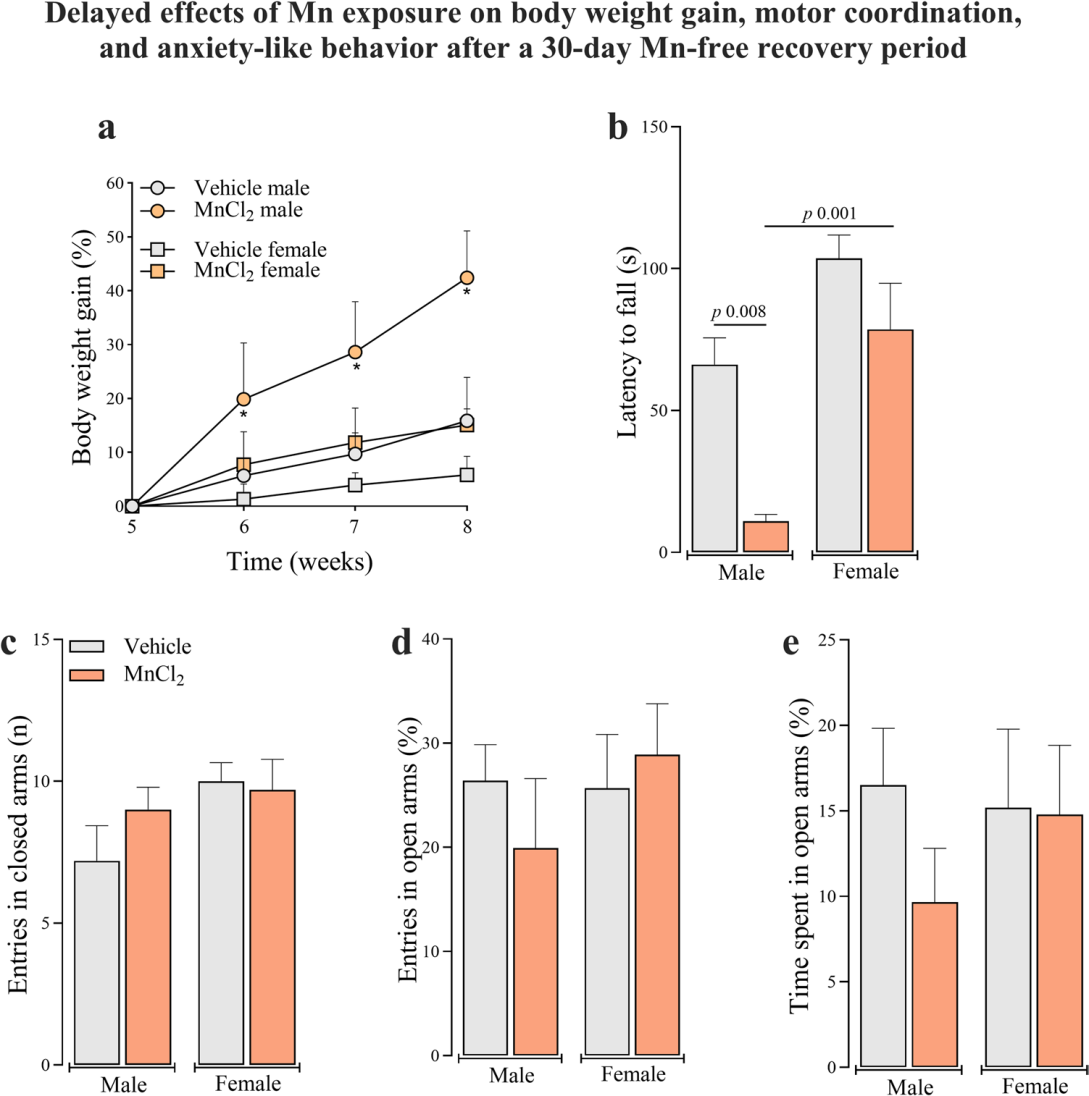
Delayed effects of subacute Mn exposure (MnCl_2_; 15 mg/kg i.p.; daily for 5 days/4 weeks) on body weight gain (**a**), motor coordination (**b**) and anxiety-like behavior (**c**-**e**) after a 30-day Mn-free recovery period in adult male and female Wistar rats. Data represent mean ± standard error of the mean (10 animals/group for body weight gain and 5 animals/group for behavioral tests). Significant *p*-values are described in each panel. Two-way ANOVA for repeated measures followed by Tukey post-hoc test was used for body weight analysis (**a**). One and Two-way ANOVA followed by Tukey post-hoc test was used for behavioral outcomes (**b**-**e**)

Considering the importance of monitoring the progression or remission of toxic effects over time, and to distinguish immediate from delayed responses following exposure cessation, behavioral parameters were evaluated in a predefined recovery cohort after and additional 30-day Mn-free period. After this recovery interval, Mn-treated animals exhibited increased body weight gain when compared to their controls in all weeks evaluated. (Fig. 5a) (interaction between group and week: [F_(1,19)_ = 21.263; *P* < 0.001], interaction between group and sex: [F_(1,19)_ = 29.063; *P* < 0.001] and interaction between group, sex and week: [F_(1,19)_ = 4.606; *P* < 0.01]).

Interestingly, after the 30-days Mn-free recovery period, Mn-treated males exhibited a shorter latency to fall compared to their controls, indicating a delayed impairment in motor coordination (Fig. 5b), whereas no difference was observed in females. Although no significant sex × group interaction was detected in the factorial two-way ANOVA model, one-way ANOVA across the four groups revealed a significant overall group effect [F_(3,16)_ = 14.26; *P* < 0.01], and post hoc comparisons confirmed that Mn-treated males performed worse than both their respective controls and Mn-treated females. In contrast, anxiety-like behaviors assessed at the delayed endpoint showed no significant differences between Mn-treated and control animals. The number of entries into the closed arms, open arms, and the time spent in the open arms of the elevated plus-maze did not differ between groups (Fig. 5c-e), indicating normalization of anxiety-related behavior following the recovery period.

**Fig. 6:**
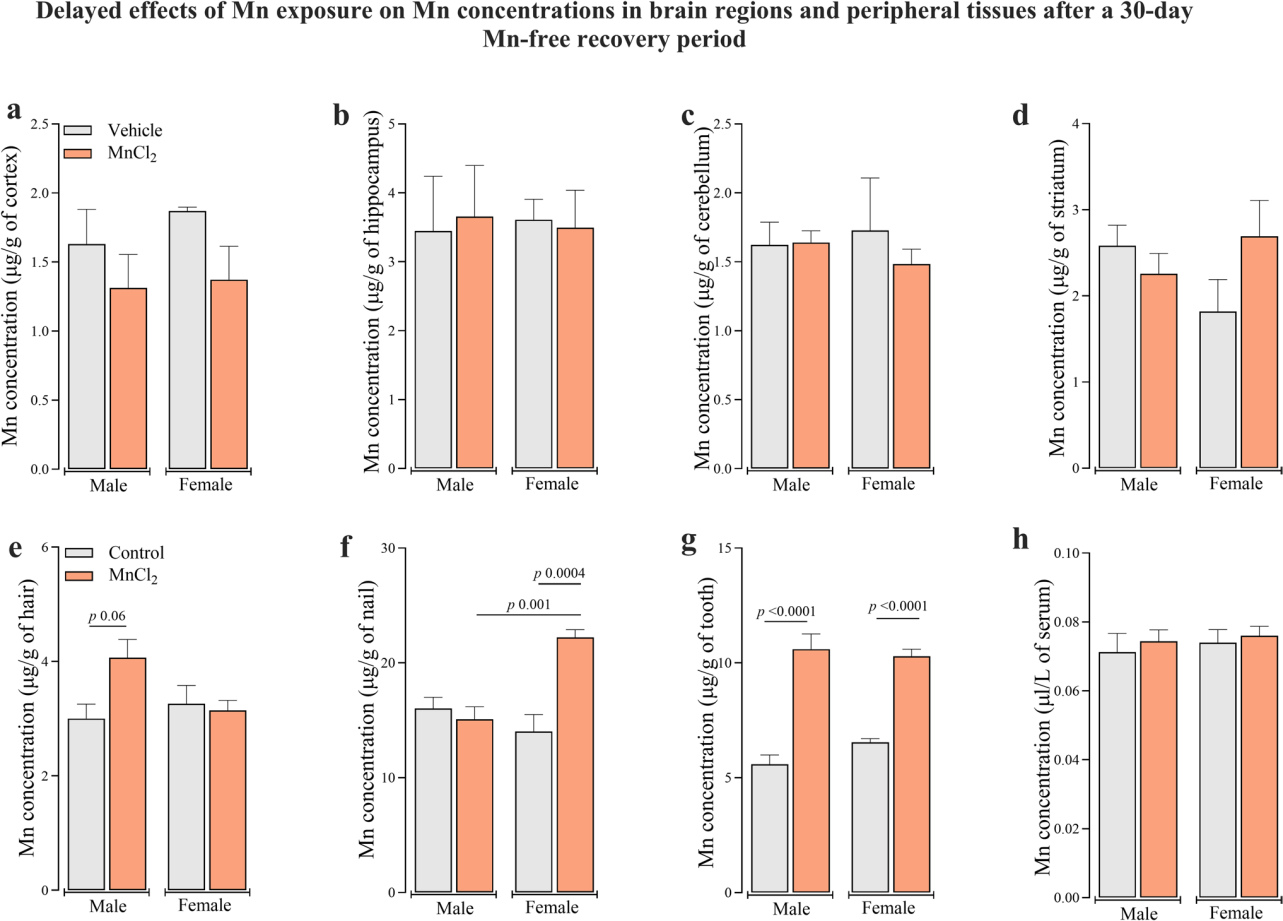
Delayed effects of subacute Mn exposure (MnCl_2_; 15 mg/kg i.p.; daily for 5 days/4 weeks) on Mn concentrations in the cerebral cortex (**a**), hippocampus (**b**), cerebellum (**c**), striatum (**d**), hair (**e**), nail (**f**), tooth (**g**) and serum (**h**) after a 30-day Mn-free recovery in adult male and female Wistar rats. Data represent mean ± standard error of the mean (5 animals/group). Significant *p*-values are described in each panel. One and Two-way ANOVA followed by Tukey post-hoc test was used for statistical comparisons

After the 30-day Mn-free recovery period, Mn levels in all brain region analyzed returned to levels comparable to control (Fig. 6a–d), indicating that the CNS no longer acted as a Mn depot at this delayed endpoint. A similar normalization pattern was observed for Fe concentrations across all brain regions (Supplementary Figure S1e-h). In contrast, at the same delayed time point, Mn concentration remained elevated in the female nails (Fig. 6f) [F_(3,16)_ = 11.29; *P* < 0.001] and in teeth of both sexes [F_(3,16)_ = 36.2; *P* < 0.001], indicating persistent retention in these keratinized tissues. Hair Mn showed a trend toward elevation after the recovery period (*p* = 0.06), although with greater inter-individual variability (Fig. 6e). Together, these findings indicate that nails and teeth provide robust markers of cumulative Mn retention, while hair may reflect exposure with greater biological variability.

Among the peripheral biomarkers assessed at the delayed endpoint, hair Mn showed the highest number of significant correlations with oxidative stress markers across multiple brain regions (Suppl. Table S3). Despite moderate correlation strength (r ranging from ± 0.30 to 0.58), this pattern suggests that hair Mn may be particularly informative of functional neurotoxic burden. Its faster growth and incorporation of circulating metals may allow it to better mirror ongoing systemic dynamics, whereas teeth and nails may reflect longer-term accumulation.

## Discussion

Mn is recognized for its neurotoxic potential, particularly affecting motor functions, mitochondrial activity, and redox homeostasis. In this study, we examined both the immediate effects observed at the end of the 30-day Mn exposure period and the delayed effects assessed after an additional 30-day Mn-free recovery interval in male and female rats. This predefined two-endpoint experimental design enabled a systematic evaluation of behavioral, biochemical, and bioaccumulation outcomes and facilitated the identification of sex-dependent vulnerabilities and reliable tissue biomarkers of Mn exposure.

Our findings demonstrated that Mn exposure produced delayed motor impairments that emerged only after a Mn-free recovery period, whereas no deficits were evident immediately after the exposure phase. This temporal dissociation suggests that compensatory neurobiological mechanisms may initially mitigate Mn neurotoxicity, but these mechanisms deteriorate over time, revealing latent dysfunction. Mn is known to accumulate preferentially in the basal ganglia structures such as the globus pallidus and substantia nigra pars reticulata, regions central to motor regulation [33], altering dopaminergic and GABAergic neurotransmission, two major pathways required for coordinated movement [8, 16, 19]. Therefore, even after Mn levels normalize in the CNS, structural and synaptic changes produced during exposure may persist and contribute to delayed motor deficits, consistent with previous evidence reporting latent Mn neurotoxicity [34]. It is important to contextualize these findings within the broader literature, which includes numerous reports describing motor deficits during or immediately after Mn exposure [12, 23, 34, 35], as well as studies showing partial recovery of motor function after exposure cessation in occupational and animal models [13, 33, 36, 37]. These studies suggest that the temporal profile of Mn-induced motor alterations varies with exposure paradigms and assessment timing, and that recovery trajectories are influenced by dose, duration, and the functional domain evaluated. In the present study, the absence of overt motor deficits immediately after exposure may reflect transient compensatory adaptations, whereas the delayed assessment captured a later window in which persistent synaptic or mitochondrial dysfunction became behaviorally evident. Thus, rather than contradicting previous work, our results align with a pattern of delayed and sex-dependent vulnerability under specific exposure and recovery conditions.

Sex-dependent biochemical changes further support differential vulnerability. Females exhibited decreased non-protein thiols in the cortex and hippocampus, indicating impaired antioxidant capacity, while mitochondrial complexes I and II displayed distinct patterns of activity modulation across sexes and brain regions. These findings align with established mechanisms of Mn toxicity involving oxidative stress and mitochondrial dysfunction [38, 39], altering antioxidant gene expression, including downregulation of SOD2, GPx, and HO-1 [38, 40], and interfering with Nrf2 signaling, a central regulator of redox homeostasis [41]. Mn can also disrupt epigenetic regulators involved in neuronal resilience and stress responses [40, 42] and impair mitochondrial biogenesis via the PGC-1α pathway, which has been implicated in Mn-associated neurodegeneration [12, 43]. Recent multi-omics studies demonstrate that Mn affects transcriptomic and proteomic networks involved in neuroinflammation, autophagy, mitophagy, and synaptic signaling [44–48], suggesting broad molecular remodeling that may persist beyond the exposure window. Moreover, Mn-induced neuroinflammation mediated by glial activation [15, 17], stimulation of TLR3/NFκB signalling [18], and NLRP3 inflammasome activation [49, 50] may contribute to long-lasting neurotoxic effects.

Although our aim was not to dissect these pathways in detail, the sex-dependent biochemical alterations observed here likely reflect differential engagement of Mn-responsive mechanisms previously described in the literature. Recent mechanistic studies support the interpretation that delayed neurochemical and behavioral alterations may arise from persistent mitochondrial and regulatory dysfunction induced by Mn. Harischandra et al. [46] demonstrated that Mn disrupts PINK1/Parkin-dependent mitophagy, promoting the accumulation of damaged mitochondria and long-term bioenergetic instability, a pattern consistent with the time-dependent modulation of mitochondrial complex I and II activities observed in our study. Complementarily, Liu et al. [47] identified Mn-induced S-nitrosylation of PINK1 as a critical upstream event impairing mitochondrial quality control, reinforcing the concept that mitochondrial dysfunction may persist even after brain Mn levels normalize. Moreover, epigenetic repression of antioxidant and mitochondrial regulatory genes, as reported by Lindner et al. [42], provides a plausible explanation for the sustained reduction in NPSH and the sex-specific metabolic vulnerability identified here. Together, these findings offer a coherent mechanistic framework linking our delayed functional outcomes to established molecular pathways of Mn neurotoxicity.

Mn-induced neurotoxicity is closely linked to disturbances in metal homeostasis, particularly through the dysregulation of Mn transporters. Among them, SLC30A10 functions as a Mn efflux transporter, limiting intracellular Mn accumulation, whereas SLC39A14 and SLC39A8 mediate Mn uptake into cells [51]. More recently, SLC39A11 has been identified as a sex-specific transporter regulating Mn metabolism and longevity, suggesting an additional layer of sex-dependent Mn regulation of homeostasis [52]. Consistent with these roles, biallelic mutations in SLC30A10 cause systemic Mn overload leading to early-onset parkinsonism, dystonia, polycythemia, and liver disease [53–55], findings further corroborated in experimental work demonstrating its essential role in biliary and intestinal Mn excretion [56]. Conversely, mutations in SLC39A14 result in severe Mn accumulation in the brain and progressive motor dysfunction, a phenotype confirmed in ZIP14-deficient mice exhibiting Mn overload and motor impairments [57], indicating that ZIP14 is necessary for regulating Mn absorption and systemic balance [58]. Taken together, these findings support the view that Mn clearance involves coordinated transporter activity, with SLC30A10-mediated efflux acting in concert with ZIP14-dependent tissue uptake [51, 57, 59].

Importantly, several Mn transporters display sex-dependent expression and regulation, which may help explain the differential susceptibility observed between male and female subjects. For instance, lower expression of SLC30A10 in females has been proposed to reduce Mn efflux capacity, favoring retention and oxidative vulnerability in regions such as the hippocampus and cortex. Moreover, polymorphisms in SLC39A8 have been linked to altered Mn transport and increased risk for neurodevelopmental and inflammatory disorders, with evidence suggesting modulation by sex hormones, where women exhibit greater Mn absorption than men [40, 59–61]. These findings align with our observation of higher Mn accumulation in females.

Although estrogen is often considered neuroprotective in Mn toxicity, our results demonstrate a more nuanced scenario. Estrogen levels fluctuate across the estrous (in rodents) or menstrual (in humans) cycle, dynamically influencing Mn uptake, distribution, and clearance. Estrogen can increase DMT1 and ZIP8 expression, enhancing Mn uptake into cells, particularly within basal ganglia [62, 63]. In humans, women show a higher prevalence of elevated Mn levels, likely due to greater Mn absorption secondary to lower iron stores, a physiological relationship also explaining the marked increase in Mn levels during pregnancy [23, 64, 65].

Furthermore, although estrogen can promote antioxidant responses, its actions are region-specific, dose-dependent, and receptor-dependent [66–68]. If receptor expression is regionally low or Mn disrupts downstream signaling pathways, estrogen´s protective effects may be attenuated or lost [36], consistent with the blunted antioxidant response observed here.

Sex-specific responses to Mn toxicity are further supported by previous work from our group. Richter Schmitz et al. (2019) demonstrated that males and females exhibit distinct oxidative and mitochondrial profiles following Mn exposure. Male rats exhibited more pronounced oxidative stress in the liver and kidney, whereas females showed increased mitochondrial complex I activity in the kidney, suggesting divergent mitochondrial adaptations or vulnerabilities across sexes [69]. Taken together, these findings indicate that Mn neurotoxicity does not confer a uniform sex bias, but rather reveals sex-dependent vulnerability across distinct biological domains, with females showing greater biochemical and metabolic sensitivity, while males exhibit more pronounced delayed functional impairment.

The delayed onset of motor impairments observed in our study underscores the importance of long-term monitoring for individuals at risk of Mn intoxication, such as workers in welding, mining, and battery manufacturing industries [70–72]. The identification of easy-to-collect, reliable, and precise biomarkers is essential for early detection and prevention of Mn-induced neurotoxicity [73–76]. In our study, hair, nails, and teeth showed elevated Mn levels after a 30-day recovery period. These tissues capture cumulative exposure and provide historical evidence of Mn accumulation that is not reflected in blood, which represents only ongoing or recent exposure [52, 75, 76]. Importantly, systematic reviews identify hair, nails, and teeth as reliable biomarkers for differentiating exposure levels in occupational and environmental settings [77]. Additionally, studies in exposed workers validate Mn in nails and hair as stable indicators of chronic Mn exposure [78]. Together, these findings emphasize the potential of nails and teeth as non-invasive, persistent biomarkers suitable for longitudinal monitoring and evaluation of interventions aimed at reducing Mn body burden. Although hair Mn did not show the same degree of statistical robustness in retention after recovery, it exhibited the strongest and most consistent correlations with oxidative stress markers. This suggests that hair may better reflect ongoing functional neurotoxic burden, whereas nails and teeth may serve as more stable indicators of cumulative exposure.

In peripheral keratinized tissues such as nails and teeth, Mn becomes incorporated during tissue formation. For nails, this process reflects a time-averaged systemic Mn exposure over weeks to months, depending on nail growth rate (79). In teeth, Mn deposition occurs during dentin and enamel mineralization, particularly during early developmental stages, where Mn may substitute for calcium or phosphate within hydroxyapatite (80). Because these tissues are metabolically inert after formation, Mn deposition is preserved and reflects historical systemic exposure. Although Mn incorporation is largely passive and concentration-dependent, systemic Mn-binding proteins and local transporter expression may influence accumulation (81). Notably, SLC30A10 expression in epithelial and exocrine tissues suggests a potential mechanism for Mn sequestration into keratinized structures [75, 77]. Although we did not identify published evidence describing sex differences in Mn incorporation into keratinized tissues, our findings suggest this possibility, given that females exhibited higher systemic Mn accumulation in several brain regions and greater overall Mn burden after exposure. These patterns raise the hypothesis that sex-dependent toxicokinetics may also influence Mn deposition in nails and teeth, which should be explored in future studies.

A key methodological consideration is the choice of intraperitoneal (i.p.) injection, which ensures precise dosing and uniform systemic bioavailability across animals. While this approach enhances experimental control, it bypasses physiologically relevant barriers associated with human exposures, such as gastrointestinal absorption and pulmonary deposition [33, 78].

Occupational exposures typically occur through inhalation, allowing Mn to reach the brain via olfactory transport and partially bypass the blood–brain barrier. In contrast, ingested Mn undergoes regulated intestinal uptake and first-pass hepatic metabolism [33, 40]. Additionally, species-specific differences also limit extrapolation to humans, as rodents absorb Mn more efficiently from the gut and differ in hepatic-biliary Mn handling compared to humans [36, 78].

Although our findings provide valuable insights into delayed Mn-induced motor deficits, potential biomarkers, and sex-specific susceptibility, some limitations must be acknowledged. The molecular pathways underlying these impairments are not fully defined and warrant further exploration, particularly regarding the reversibility of Mn-induced neurotoxicity and the identification of targeted therapeutic strategies. Additionally, biochemical parameters were not assessed after the 30-day recovery period because the study was primarily designed to evaluate behavioral outcomes and identify peripheral biomarkers of cumulative Mn exposure. Moreover, the amount of available biological material after tissue allocation for the planned analyses was insufficient to reliable perform the additional biochemical assays across all brain regions. Despite these considerations, our study demonstrates clear sex-dependent effects of Mn on oxidative stress, mitochondrial function, and behavior. Furthermore, the identification of nails and teeth as promising biomarkers provides new opportunities for non-invasive monitoring of cumulative Mn exposure, with particular relevance for at-risk occupational populations such as welders, miners, and battery workers.

## Conclusions

This study demonstrates that Mn exposure induces delayed motor impairments associated with oxidative stress and mitochondrial dysfunction, exhibiting distinct sex-dependent effects. Female rats were more susceptible to oxidative and mitochondrial dysfunction, whereas males showed greater impairments in motor coordination after the recovery period. In addition, peripheral keratinized tissues, particularly nails and teeth, emerged as promising non-invasive biomarkers for monitoring cumulative Mn exposure, while hair Mn showed stronger associations with biochemical alterations. Overall, our findings support a pattern of Mn neurotoxicity characterized by sex-dependent vulnerability rather than a uniform susceptibility, with females displaying greater biochemical sensitivity and males exhibiting more pronounced delayed functional impairment.

## Supplementary Information

Below is the link to the electronic supplementary material.

ESM 1 (DOCX 778 KB)

## Data Availability

The datasets generated during and/or analyzed during the current study are available from the corresponding author on reasonable request.

## References

[1] Balachandran RC, Mukhopadhyay S, McBride D, Veevers J, Harrison FE, Aschner M et al (2020) Brain manganese and the balance between essential roles and neurotoxicity. J Biol Chem 295(19):6312–29. 10.1074/jbc.REV119.009453 32188696 10.1074/jbc.REV119.009453 PMC7212623

[2] Peres TV, Schettinger MRC, Chen P, Carvalho F, Avila DS, Bowman AB et al (2016) Manganese-induced neurotoxicity: a review of its behavioral consequences and neuroprotective strategies. BMC Pharmacol Toxicol 17(1):57. 10.1186/s40360-016-0099-0 27814772 10.1186/s40360-016-0099-0 PMC5097420

[3] WHO WHO. Manganese in drinking water: background document for development of WHO guidelines for drinking-water quality. [Internet]. 2021 [cited 2025 Apr 9]. Located at: https://www.who.int/docs/default-source/wash-documents/wash-chemicals/manganese-background-document.pdf. Available from: https://www.who.int/docs/default-source/wash-documents/wash-chemicals/manganese-background-document.pdf

[4] Kornblith ES, Casey SL, Lobdell DT, Colledge MA, Bowler RM (2018) Environmental exposure to manganese in air: tremor, motor and cognitive symptom profiles. Neurotoxicology 64:152–158. 10.1016/j.neuro.2017.09.012 28965701 10.1016/j.neuro.2017.09.012 PMC6260785

[5] Mergler D, Baldwin M, Bélanger S, Larribe F, Beuter A, Bowler R et al (1999) Manganese neurotoxicity, a continuum of dysfunction: results from a community based study. Neurotoxicology 20(2–3):327–342 (**PubMed PMID: 10385894**) 10385894

[6] Schullehner J, Thygesen M, Kristiansen SM, Hansen B, Pedersen CB, Dalsgaard S (2020) Exposure to manganese in drinking water during childhood and association with attention-deficit hyperactivity disorder: a nationwide cohort study. Environ Health Perspect 128(9):97004. 10.1289/EHP6391 32955354 10.1289/EHP6391 PMC7505135

[7] Liu X, Sullivan KA, Madl JE, Legare M, Tjalkens RB (2006) Manganese-induced neurotoxicity: the role of astroglial-derived nitric oxide in striatal interneuron degeneration. Toxicol Sci 91(2):521–531. 10.1093/toxsci/kfj150 16551646 10.1093/toxsci/kfj150

[8] Bouabid S, Tinakoua A, Lakhdar-Ghazal N, Benazzouz A (2016) Manganese neurotoxicity: behavioral disorders associated with dysfunctions in the basal ganglia and neurochemical transmission. J Neurochem 136(4):677–691. 10.1111/jnc.13442 26608821 10.1111/jnc.13442

[9] Amos-Kroohs RM, Usach V, Piñero G, Vorhees CV, Martinez Vivot R, Soto PA et al (2019) Metal bashing: iron deficiency and manganese overexposure impact on peripheral nerves. J Toxicol Environ Health A 82(2):99–112. 10.1080/15287394.2019.1566105 30652531 10.1080/15287394.2019.1566105 PMC6397089

[10] Viana GFdeS, de Carvalho CF, Nunes LS, Rodrigues JLG, Ribeiro NS, de Almeida DA et al (2014) Noninvasive biomarkers of manganese exposure and neuropsychological effects in environmentally exposed adults in Brazil. Toxicol Lett 231(2):169–178. 10.1016/j.toxlet.2014.06.018 24992226 10.1016/j.toxlet.2014.06.018

[11] Martin KV, Edmondson D, Cecil KM, Bezi C, Vance ML, McBride D et al (2020) Manganese exposure and neurologic outcomes in adult populations. Neurol Clin 38(4):913–936. 10.1016/j.ncl.2020.07.008 33040869 10.1016/j.ncl.2020.07.008 PMC8978550

[12] Lang J, Gao L, Wu J, Meng J, Gao X, Ma H et al (2022) Resveratrol attenuated manganese-induced learning and memory impairments in mice through PGC-1Alpha-mediated autophagy and microglial M1/M2 polarization. Neurochem Res 47(11):3414–27. 10.1007/s11064-022-03695-w 35871432 10.1007/s11064-022-03695-w

[13] Pajarillo E, Demayo M, Digman A, Nyarko-Danquah I, Son DS, Aschner M et al (2022) Deletion of RE1-silencing transcription factor in striatal astrocytes exacerbates manganese-induced neurotoxicity in mice. Glia 70(10):1886–901. 10.1002/glia.24226 35638297 10.1002/glia.24226 PMC9378447

[14] Richter Schmitz CR, Eichwald T, Branco Flores MV, Varela KG, Mantovani A, Steffani JA et al (2019) Sex differences in subacute manganese intoxication: oxidative parameters and metal deposition in peripheral organs of adult Wistar rats. Regul Toxicol Pharmacol 104:98–107. 10.1016/j.yrtph.2019.03.005 30878574 10.1016/j.yrtph.2019.03.005

[15] Soto-Verdugo J, Ortega A (2021) Critical involvement of glial cells in manganese neurotoxicity. BioMed Res Int 2021(1):1596185. 10.1155/2021/1596185 34660781 10.1155/2021/1596185 PMC8514895

[16] Sidoryk-Wegrzynowicz M, Aschner M (2013) Manganese toxicity in the central nervous system: the glutamine/glutamate-γ-aminobutyric acid cycle. J Intern Med 273(5):466–477. 10.1111/joim.12040 23360507 10.1111/joim.12040 PMC3633698

[17] Kirkley KS, Popichak KA, Afzali MF, Legare ME, Tjalkens RB (2017) Microglia amplify inflammatory activation of astrocytes in manganese neurotoxicity. J Neuroinflammation 14(1):99. 10.1186/s12974-017-0871-0 28476157 10.1186/s12974-017-0871-0 PMC5418760

[18] Gokhale A, Mendez-Vazquez H, Sampson MM, Moctezuma FGR, Harbuzariu A, Sing A, et al. Mitochondrially Transcribed dsRNA Mediates Manganese-induced Neuroinflammation. BioRxiv Prepr Serv Biol. 2025 Feb 20;2025.02.16.638529. 10.1101/2025.02.16.638529 PubMed PMID: 40027638; PubMed Central PMCID: PMC11870567.

[19] Marwah PK, Paik G, Issa CJ, Jemison CC, Qureshi MB, Hanna TM et al (2022) Manganese-stimulated redox cycling of dopamine derivatives: implications for manganism. Neurotoxicology 90:10–8. 10.1016/j.neuro.2022.02.007 35217070 10.1016/j.neuro.2022.02.007

[20] Erikson KM, Thompson K, Aschner J, Aschner M (2007) Manganese neurotoxicity: a focus on the neonate. Pharmacol Ther 113(2):369–377. 10.1016/j.pharmthera.2006.09.002 17084903 10.1016/j.pharmthera.2006.09.002 PMC1852452

[21] Scholefield M, Unwin RD, Cooper GJS (2020) Shared perturbations in the metallome and metabolome of Alzheimer’s, Parkinson’s, Huntington’s, and dementia with Lewy bodies: a systematic review. Ageing Res Rev 63:101152. 10.1016/j.arr.2020.101152 32846222 10.1016/j.arr.2020.101152

[22] Nakagawa Y, Yamada S (2020) Metal homeostasis disturbances in neurodegenerative disorders, with special emphasis on Creutzfeldt-Jakob disease - potential pathogenetic mechanism and therapeutic implications. Pharmacol Ther 207:107455. 10.1016/j.pharmthera.2019.107455 31863817 10.1016/j.pharmthera.2019.107455

[23] O’Neal SL, Lee JW, Zheng W, Cannon JR (2014) Subacute manganese exposure in rats is a neurochemical model of early manganese toxicity. Neurotoxicology 44:303–313. 10.1016/j.neuro.2014.08.001 25117542 10.1016/j.neuro.2014.08.001 PMC4278355

[24] Pellow S, Chopin P, File SE, Briley M (1985) Validation of open : closed arm entries in an elevated plus-maze as a measure of anxiety in the rat. J Neurosci Methods 14(3):149–167. 10.1016/0165-0270(85)90031-7 2864480 10.1016/0165-0270(85)90031-7

[25] Jiang C, Wan X, Jankovic J, Christian ST, Pristupa ZB, Niznik HB et al (2004) Dopaminergic properties and experimental anti-Parkinsonian effects of IPX750 in rodent models of Parkinson disease. Clin Neuropharmacol 27(2):63–73. 10.1097/00002826-200403000-00004 15252266 10.1097/00002826-200403000-00004

[26] Fitsanakis VA, Zhang N, Anderson JG, Erikson KM, Avison MJ, Gore JC et al (2008) Measuring brain manganese and iron accumulation in rats following 14 weeks of low-dose manganese treatment using atomic absorption spectroscopy and magnetic resonance imaging. Toxicol Sci 103(1):116–24. 10.1093/toxsci/kfn019 18234737 10.1093/toxsci/kfn019 PMC7910808

[27] Esterbauer H, Cheeseman KH. [42] Determination of aldehydic lipid peroxidation products: Malonaldehyde and 4-hydroxynonenal. In. 1990. p. 407–21. 10.1016/0076-6879(90)86134-H 10.1016/0076-6879(90)86134-h 2233308

[28] Ellman GL (1959) Tissue sulfhydryl groups. Arch Biochem Biophys 82(1):70–77. 10.1016/0003-9861(59)90090-6 13650640 10.1016/0003-9861(59)90090-6

[29] Latini A, Rodriguez M, Borba Rosa R, Scussiato K, Leipnitz G, Reis de Assis D et al (2005) 3-hydroxyglutaric acid moderately impairs energy metabolism in brain of young rats. Neuroscience 135(1):111–20. 10.1016/j.neuroscience.2005.05.013 16111821 10.1016/j.neuroscience.2005.05.013

[30] Cassina A, Radi R (1996) Differential inhibitory action of nitric oxide and peroxynitrite on mitochondrial electron transport. Arch Biochem Biophys 328(2):309–316. 10.1006/abbi.1996.0178 8645009 10.1006/abbi.1996.0178

[31] Fischer JC, Ruitenbeek W, Berden JA, Trijbels JMF, Veerkamp JH, Stadhouders AM et al (1985) Differential investigation of the capacity of succinate oxidation in human skeletal muscle. Clin Chim Acta 153(1):23–36. 10.1016/0009-8981(85)90135-4 3000647 10.1016/0009-8981(85)90135-4

[32] Lowry OH, Rosebrough NJ, Farr AL, Randall RJ. Protein measurement with the folin phenol reagent. Anal Biochem. 1951;265–75. 10.1016/0304-3894(92)87011-4 PubMed PMID: 14907713. 14907713

[33] Aschner M, Erikson KM, Dorman DC (2005) Manganese dosimetry: species differences and implications for neurotoxicity. Crit Rev Toxicol 35(1):1–32. 10.1080/10408440590905920 15742901 10.1080/10408440590905920

[34] Stanwood GD, Leitch DB, Savchenko V, Wu J, Fitsanakis VA, Anderson DJ et al (2009) Manganese exposure is cytotoxic and alters dopaminergic and GABAergic neurons within the basal ganglia. J Neurochem 110(1):378–89. 10.1111/j.1471-4159.2009.06145.x 19457100 10.1111/j.1471-4159.2009.06145.x PMC2737271

[35] Kim S, Pajarillo E, Digman A, Ajayi I, Son DS, Aschner M et al (2025) Role of dopaminergic RE1-silencing transcription factor (REST) in manganese-induced behavioral deficits and dysregulating dopaminergic and serotonergic neurotransmission in mice. Neurotoxicology 108:57–68. 10.1016/j.neuro.2025.03.001 40057281 10.1016/j.neuro.2025.03.001 PMC12983288

[36] Fitsanakis VA, Zhang N, Avison MJ, Gore JC, Aschner JL, Aschner M (2006) The use of magnetic resonance imaging (MRI) in the study of manganese neurotoxicity. Neurotoxicology. 10.1016/j.neuro.2006.03.001 10.1016/j.neuro.2006.03.001 16620989

[37] Evans GR, Masullo LN. Manganese Toxicity. In: StatPearls [Internet]. Treasure Island (FL): StatPearls Publishing; 2025 [cited 2026 Feb 27]. Available from: http://www.ncbi.nlm.nih.gov/books/NBK560903/ PubMed PMID: 32809738. 32809738

[38] Martinez-Finley EJ, Gavin CE, Aschner M, Gunter TE (2013) Manganese neurotoxicity and the role of reactive oxygen species. Free Radic Biol Med 62:65–75. 10.1016/j.freeradbiomed.2013.01.032 23395780 10.1016/j.freeradbiomed.2013.01.032 PMC3713115

[39] Martins AC, Oliveira-Paula GH, Tinkov AA, Skalny AV, Tizabi Y, Bowman AB et al (2025) Role of manganese in brain health and disease: focus on oxidative stress. Free Radic Biol Med 232:306–18. 10.1016/j.freeradbiomed.2025.03.013 40086492 10.1016/j.freeradbiomed.2025.03.013 PMC11985276

[40] Tinkov AA, Paoliello MMB, Mazilina AN, Skalny AV, Martins AC, Voskresenskaya ON et al (2021) Molecular targets of manganese-induced neurotoxicity: a five-year update. Int J Mol Sci 22(9):4646. 10.3390/ijms22094646 33925013 10.3390/ijms22094646 PMC8124173

[41] Chen P, Totten M, Zhang Z, Bucinca H, Erikson K, Santamaría A et al (2019) Iron and manganese-related CNS toxicity: mechanisms, diagnosis and treatment. Expert Rev Neurother 19(3):243–60. 10.1080/14737175.2019.1581608 30759034 10.1080/14737175.2019.1581608 PMC6422746

[42] Lindner S, Lucchini R, Broberg K (2022) Genetics and epigenetics of manganese toxicity. Curr Environ Health Rep 9(4):697–713. 10.1007/s40572-022-00384-2 36357556 10.1007/s40572-022-00384-2 PMC9729127

[43] Piccinin E, Sardanelli AM, Seibel P, Moschetta A, Cocco T, Villani G (2021) PGC-1s in the spotlight with Parkinson’s disease. Int J Mol Sci 22(7):3487. 10.3390/ijms22073487 33800548 10.3390/ijms22073487 PMC8036867

[44] Zhang S, Wu L, Zhang J, Wang X, Yang X, Xin Y et al (2023) Multi-omics analysis reveals Mn exposure affects ferroptosis pathway in zebrafish brain. Ecotoxicol Environ Saf 253:114616. 10.1016/j.ecoenv.2023.114616 36796209 10.1016/j.ecoenv.2023.114616

[45] Fernandes J, Chandler JD, Lili LN, Uppal K, Hu X, Hao L et al (2019) Transcriptome analysis reveals distinct responses to physiologic versus toxic manganese exposure in human neuroblastoma cells. Front Genet. 10.3389/fgene.2019.00676 31396262 10.3389/fgene.2019.00676 PMC6668488

[46] Harischandra DS, Ghaisas S, Zenitsky G, Jin H, Kanthasamy A, Anantharam V et al (2019) Manganese-induced neurotoxicity: new insights into the triad of protein misfolding, mitochondrial impairment, and neuroinflammation. Front Neurosci 13:654. 10.3389/fnins.2019.00654 31293375 10.3389/fnins.2019.00654 PMC6606738

[47] Liu K, Liu Z, Liu Z, Ma Z, Deng Y, Liu W et al (2022) Manganese induces S-nitrosylation of PINK1 leading to nerve cell damage by repressing PINK1/Parkin-mediated mitophagy. Sci Total Environ 834:155358. 10.1016/j.scitotenv.2022.155358 35460769 10.1016/j.scitotenv.2022.155358

[48] Yan DY, Xu B (2020) The role of autophagy in manganese-induced neurotoxicity. Front Neurosci 14:574750. 10.3389/fnins.2020.574750 33041767 10.3389/fnins.2020.574750 PMC7522436

[49] Singh S, Shaikh IA, More SS, Mahnashi MH, Almohaimeed HM, El-Sherbiny M et al (2022) Blockage of KHSRP-NLRP3 by MCC950 can reverse the effect of manganese-induced neuroinflammation in N2a cells and rat brain. Int J Mol Sci 23(21):13224. 10.3390/ijms232113224 36362011 10.3390/ijms232113224 PMC9658363

[50] Sarkar S, Rokad D, Malovic E, Luo J, Harischandra DS, Jin H et al (2019) Manganese activates NLRP3 inflammasome signaling and propagates exosomal release of ASC in microglial cells. Sci Signal 12(563):eaat9900. 10.1126/scisignal.aat9900 30622196 10.1126/scisignal.aat9900 PMC6420319

[51] Kapoor D, Garg D, Sharma S, Goyal V (2021) Inherited manganese disorders and the brain: what neurologists need to know. Ann Indian Acad Neurol 24(1):15–21. 10.4103/aian.AIAN_789_20 33911374 10.4103/aian.AIAN_789_20 PMC8061520

[52] Deng X, Guo Y, Jin X, Si H, Dai K, Deng M et al (2024) Manganese accumulation in red blood cells as a biomarker of manganese exposure and neurotoxicity. Neurotoxicology 102:1–11. 10.1016/j.neuro.2024.03.003 38461971 10.1016/j.neuro.2024.03.003

[53] Tuschl K, Meyer E, Valdivia LE, Zhao N, Dadswell C, Abdul-Sada A et al (2016) Mutations in SLC39A14 disrupt manganese homeostasis and cause childhood-onset parkinsonism-dystonia. Nat Commun 7(1):11601. 10.1038/ncomms11601 27231142 10.1038/ncomms11601 PMC4894980

[54] Xia Z, Wei J, Li Y, Wang J, Li W, Wang K et al (2017) Zebrafish slc30a10 deficiency revealed a novel compensatory mechanism of Atp2c1 in maintaining manganese homeostasis. PLoS Genet 13(7):e1006892. 10.1371/journal.pgen.1006892 28692648 10.1371/journal.pgen.1006892 PMC5524415

[55] Mercadante CJ, Prajapati M, Conboy HL, Dash ME, Herrera C, Pettiglio MA et al (2019) Manganese transporter Slc30a10 controls physiological manganese excretion and toxicity. J Clin Invest 129(12):5442–5461. 10.1172/JCI129710 31527311 10.1172/JCI129710 PMC6877324

[56] Aydemir TB, Kim MH, Kim J, Colon-Perez LM, Banan G, Mareci TH et al (2017) Metal transporter Zip14 (Slc39a14) deletion in mice increases manganese deposition and produces neurotoxic signatures and diminished motor activity. J Neurosci 37(25):5996–6006. 10.1523/JNEUROSCI.0285-17.2017 28536273 10.1523/JNEUROSCI.0285-17.2017 PMC5481939

[57] Scheiber IF, Wu Y, Morgan SE, Zhao N (2019) The intestinal metal transporter ZIP14 maintains systemic manganese homeostasis. J Biol Chem 294(23):9147–9160. 10.1074/jbc.RA119.008762 31028174 10.1074/jbc.RA119.008762 PMC6556583

[58] Lucchini RG, Aschner M, Landrigan PJ, Cranmer JM (2018) Neurotoxicity of manganese: indications for future research and public health intervention from the Manganese 2016 conference. Neurotoxicology 64:1–4. 10.1016/j.neuro.2018.01.002 29429640 10.1016/j.neuro.2018.01.002 PMC6058309

[59] Xia Z, Tang B, Li X, Li X, Jia Y, Jiang J et al (2024) A novel role for the longevity-associated protein SLC39A11 as a manganese transporter. Research 7:0440. 10.34133/research.0440 39114488 10.34133/research.0440 PMC11304475

[60] Budinger D, Barral S, Soo AKS, Kurian MA (2021) The role of manganese dysregulation in neurological disease: emerging evidence. Lancet Neurol 20(11):956–968. 10.1016/S1474-4422(21)00238-6 34687639 10.1016/S1474-4422(21)00238-6

[61] Huang E, Ong WY, Connor JR (2004) Distribution of divalent metal transporter-1 in the monkey basal ganglia. Neuroscience 128(3):487–496. 10.1016/j.neuroscience.2004.06.055 15381278 10.1016/j.neuroscience.2004.06.055

[62] Morgan SE, Schroten H, Ishikawa H, Zhao N (2020) Localization of ZIP14 and ZIP8 in HIBCPP cells. Brain Sci 10(8):8. 10.3390/brainsci10080534 10.3390/brainsci10080534 PMC7464652 32784388

[63] Lee JW, Lee CK, Moon CS, Choi IJ, Lee KJ, Yi SM et al (2012) Korea National Survey for Environmental Pollutants in the Human Body 2008: heavy metals in the blood or urine of the Korean population. Int J Hyg Environ Health. 10.1016/j.ijheh.2012.01.002 10.1016/j.ijheh.2012.01.002 22341685

[64] Oulhote Y, Mergler D, Bouchard MF (2014) Sex- and age-differences in blood manganese levels in the U.S. general population: national health and nutrition examination survey 2011–2012. Environ Health 65(1):87. 10.1186/1476-069X-13-87 10.1186/1476-069X-13-87 PMC4271487 25342305

[65] Osterlund MK, Grandien K, Keller E, Hurd YL (2000) The human brain has distinct regional expression patterns of estrogen receptor alpha mRNA isoforms derived from alternative promoters. J Neurochem 75(4):1390–1397. 10.1046/j.1471-4159.2000.0751390.x 10987818 10.1046/j.1471-4159.2000.0751390.x

[66] Lee ESY, Sidoryk M, Jiang H, Yin Z, Aschner M (2009) Estrogen and tamoxifen reverse manganese-induced glutamate transporter impairment in astrocytes. J Neurochem 110(2):530–544. 10.1111/j.1471-4159.2009.06105.x 19453300 10.1111/j.1471-4159.2009.06105.x PMC3920654

[67] Digman A, Pajarillo E, Kim S, Ajayi I, Son DS, Aschner M et al (2025) Tamoxifen induces protection against manganese toxicity by REST upregulation via the ER-α/Wnt/β-catenin pathway in neuronal cells. J Biol Chem (6):108529. 10.1016/j.jbc.2025.108529 40280417 10.1016/j.jbc.2025.108529 PMC12152632

[68] Pyatha S, Kim H, Lee D, Kim K (2022) Association between heavy metal exposure and Parkinson’s disease: a review of the mechanisms related to oxidative stress. Antioxidants (Basel) 71(12):2467. 10.3390/antiox11122467 10.3390/antiox11122467 PMC9774122 36552676

[69] Nadig APR, Huwaimel B, Alobaida A, Khafagy ES, Alotaibi HF, Moin A et al (2022Nov) Manganese chloride (MnCl2) induced novel model of Parkinson’s disease in adult Zebrafish; Involvement of oxidative stress, neuroinflammation and apoptosis pathway. Biomed Pharmacother Biomedecine Pharmacother 155:113697. 10.1016/j.biopha.2022.113697. (**PubMed PMID: 36137406**) 10.1016/j.biopha.2022.113697 36137406

[70] Zheng W, Fu SX, Dydak U, Cowan DM (2011) Biomarkers of manganese intoxication. Neurotoxicology 32(1):1–8. 10.1016/j.neuro.2010.10.002 20946915 10.1016/j.neuro.2010.10.002 PMC3030659

[71] Baker MG, Simpson CD, Sheppard L, Stover B, Morton J, Cocker J et al (2015) Variance components of short-term biomarkers of manganese exposure in an inception cohort of welding trainees. J Trace Elem Med Biol 29:123–9. 10.1016/j.jtemb.2014.05.004 24916793 10.1016/j.jtemb.2014.05.004 PMC4241381

[72] Karyakina NA, Shilnikova N, Farhat N, Ramoju S, Cline B, Momoli F et al (2022) Biomarkers for occupational manganese exposure. Crit Rev Toxicol 52(8):636–663. 10.1080/10408444.2022.2128718 36705643 10.1080/10408444.2022.2128718

[73] Lotah HNA, Agarwal AK, Khanam R (2022) Heavy metals in hair and nails as markers of occupational hazard among welders working in United Arab Emirates. Toxicol Res 38(1):63–68. 10.1007/s43188-021-00091-4 35070942 10.1007/s43188-021-00091-4 PMC8748598

[74] Laohaudomchok W, Lin X, Herrick RF, Fang SC, Cavallari JM, Christiani DC et al (2011) Toenail, blood, and urine as biomarkers of manganese exposure. J Occup Environ Med 53(5):506–10. 10.1097/JOM.0b013e31821854da 21494156 10.1097/JOM.0b013e31821854da PMC3092003

[75] Arora M, Bradman A, Austin C, Vedar M, Holland N, Eskenazi B et al (2012) Determining fetal manganese exposure from mantle dentine of deciduous teeth. Environ Sci Technol 46(9):5118–25. 10.1021/es203569f 22455722 10.1021/es203569f PMC3341525

[76] Roth J, Ponzoni S, Aschner M (2013) Manganese homeostasis and transport. Met Ions Life Sci 12:169–201. 10.1007/978-94-007-5561-1_6PubMedPMID:23595673;PubMedCentralPMCID:PMC6542352 23595673 10.1007/978-94-007-5561-1_6 PMC6542352

[77] Shilnikova N, Karyakina N, Farhat N, Ramoju S, Cline B, Momoli F et al (2022) Biomarkers of environmental manganese exposure. Crit Rev Toxicol 52(4):325–43. 10.1080/10408444.2022.2095979 35894753 10.1080/10408444.2022.2095979

[78] Dorman DC, Struve MF, Marshall MW, Parkinson CU, James RA, Wong BA. Tissue manganese concentrations in young male rhesus monkeys following subchronic manganese sulfate inhalation. Toxicol Sci Off J Soc Toxicol. 2006 Jul;92(1):201–10.doi:10.1093/toxsci/kfj206 PubMed PMID: 16624849. 10.1093/toxsci/kfj206 16624849

