# Supplementary material for: Manganese Exposure: Delayed Effects and Biomarkers in adult male and female Wistar rats
Source: Mol Neurobiol. 2026 Mar 30;63(1):536. doi: 10.1007/s12035-026-05812-0 (PMC13035577; doi:10.1007/s12035-026-05812-0)
Supplement: Supplementary file 1 — (DOCX 778 KB) [file 12035_2026_5812_MOESM1_ESM.docx]

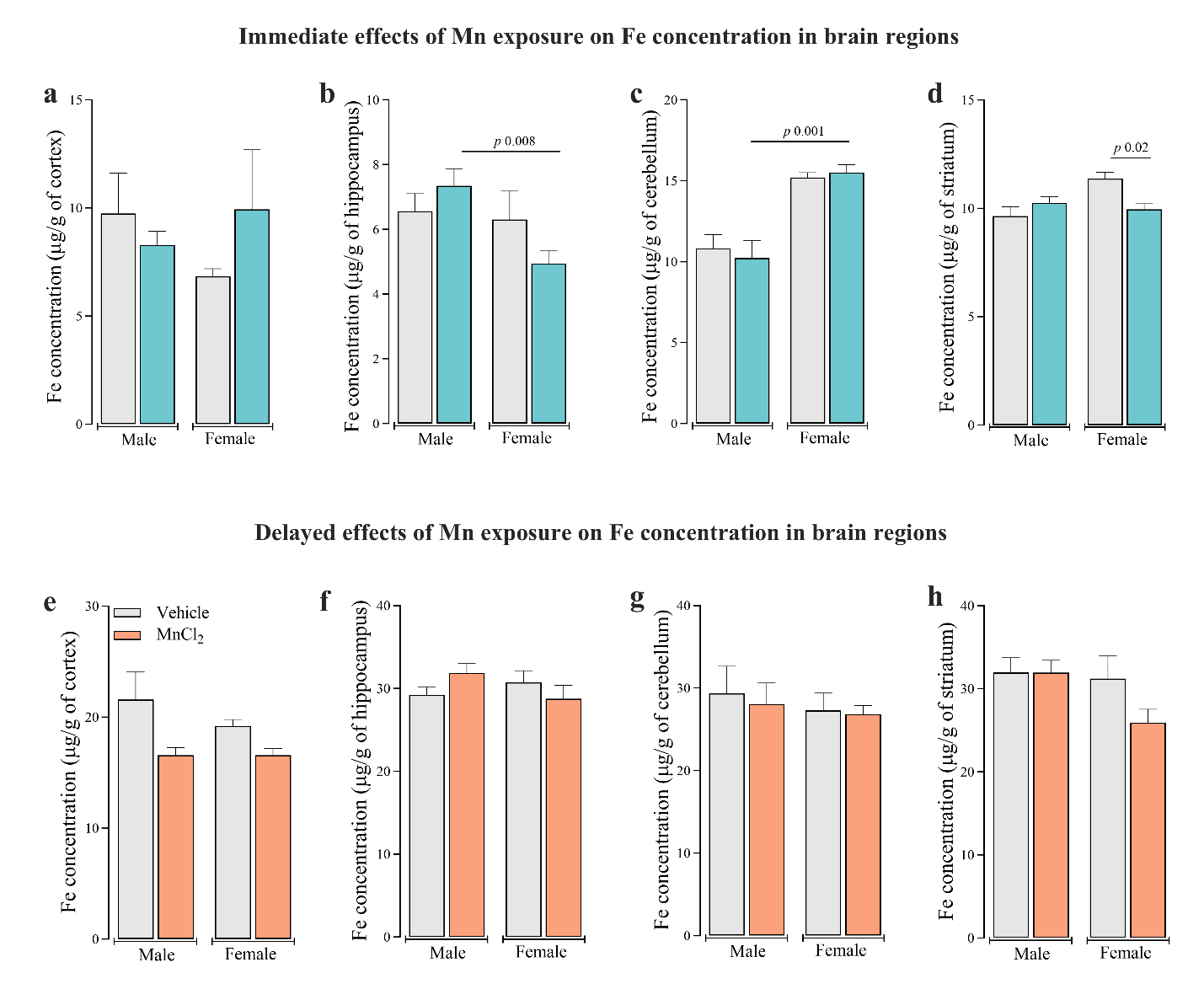


**Supplementary Figure 1.** Iron concentration in brain regions of adult male and female rats evaluated at the immediate post-exposure endpoint (MnCl_2_; 15 mg/kg i.p.; daily for 5 days/4 weeks) and after a 30-day Mn-free recovery period in cerebral cortex (a and e), hippocampus (b and f), cerebellum (c and g) and striatum (d and h), respectively. Data represent mean ± standard error of the mean mean (7 animals/group for immediate post-exposure endpoint and 5 animals/group after a 30-day Mn-free recovery period). Significant p-values and interaction effects are indicated in each panel. One and Two-way ANOVA followed by Tukey post-hoc test was used for statistical comparisons.

**Supplementary Table S1.** Both experimental cohorts (immediate and recovery) initially started with n = 10 animals per sex per group. Body weight measurements were obtained from all animals (n = 10 per sex per group) in both cohorts. For the immediate (30-day exposure) cohort, behavioral testing (Rotarod and EPM) was conducted in n = 6 animals per sex per group, with the remaining animals excluded based on predefined technical criteria described in the Methods section. For the recovery (60-day) cohort, behavioral and biochemical analyses were conducted in n = 5 animals per sex per group, while the remaining animals were allocated to an independent experimental protocol not included in the present study. For biochemical and oxidative assays in the immediate cohort, tissue allocation followed predefined methodological requirements, since each analytical technique required distinct sample preparation procedures, preventing all assays from being performed in every animal.


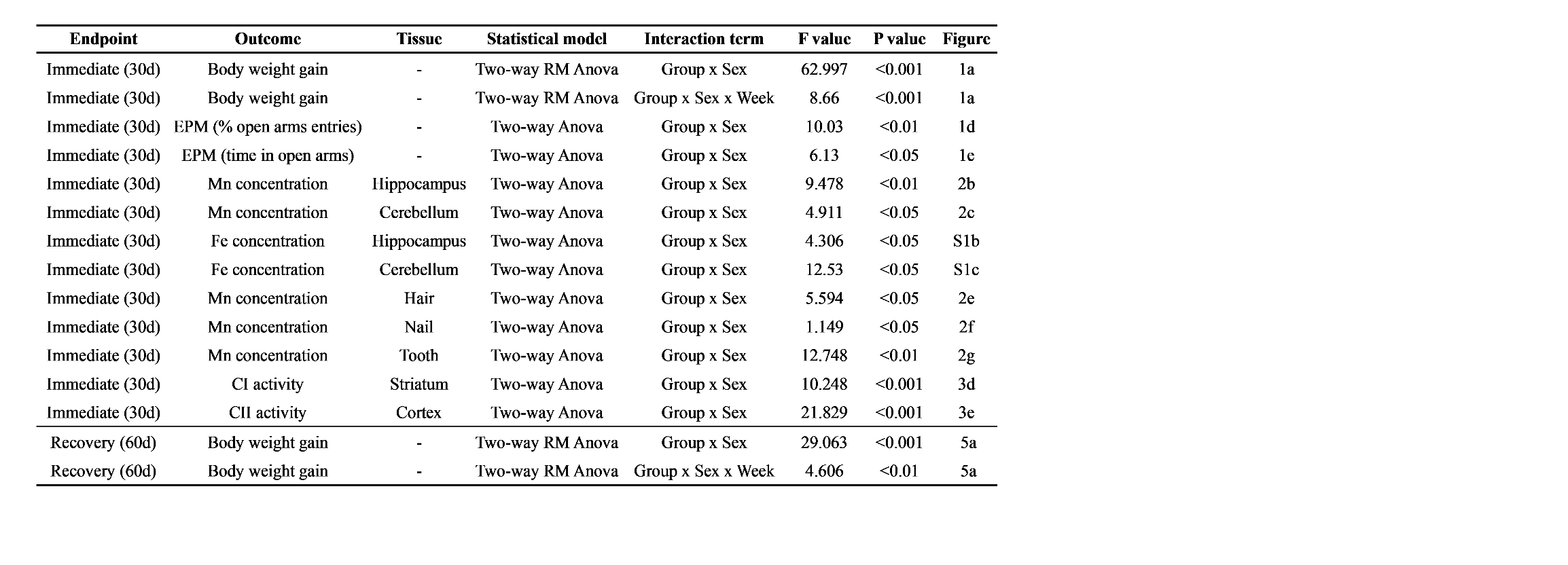


**Supplementary Table S2.** Summary of significant sex-related interactions across experimental endpoints. Statistically significant interactions involving sex (sex × group and, when applicable, sex × group × week) identified in the immediate (30-day) and recovery (60-day) cohorts are presented, together with the corresponding statistical model (two-way ANOVA or repeated-measures (RM) two-way ANOVA), interaction term, F value, p value, and figure reference.

**Supplementary Table S3.** Pearson’s correlation coefficients between manganese (Mn) concentrations in hair, tooth, and nail and oxidative stress variables in distinct brain regions of adult male and female Wistar rats following Mn exposure. Peripheral tissues were evaluated as potential non-invasive biomarkers of delayed neurotoxic effects.
